# Supplementary material for: Resolving the full spectrum of human genome variation using Linked-Reads
Source: Genome Res. 2019 Apr;29(4):635–45. doi: 10.1101/gr.234443.118 (PMC6442396; doi:10.1101/gr.234443.118)
Supplement: Supplemental Material [file supp_gr.234443.118_Supplemental_longranger-2.2.zip › longranger-2.2.tar.bz2!/lr2.2-public/lib/stan/cmdstan/stan/doc/api/html/classstan_1_1json_1_1json__handler.html]

Stan: stan::json::json\_handler Class Reference


|  |  |
| --- | --- |
|  | Stan  2.5.0  probability, sampling & optimization |


- Main Page
- Related Pages
- Namespaces
- Classes
- Files

- Class List
- Class Index
- Class Hierarchy
- Class Members

All Classes Namespaces Files Functions Variables Typedefs Enumerations Enumerator Friends Macros Pages

- stan
- json
- json\_handler

Public Member Functions |
List of all members

stan::json::json\_handler Class Reference

Abstract base class for JSON handlers.
More...

`#include <json_handler.hpp>`

Inheritance diagram for stan::json::json\_handler:

|  |  |
| --- | --- |
| Public Member Functions | |
|  | json\_handler () |
|  | |
|  | ~json\_handler () |
|  | |
| virtual void | start\_text () |
|  | Handle the the start of the text. More... |
|  | |
| virtual void | end\_text () |
|  | Handle the the end of the text. More... |
|  | |
| virtual void | start\_array () |
|  | Handle the start of an array. More... |
|  | |
| virtual void | end\_array () |
|  | Handle the end of an array. More... |
|  | |
| virtual void | start\_object () |
|  | Handle the start of an object. More... |
|  | |
| virtual void | end\_object () |
|  | Handle the end of an object. More... |
|  | |
| virtual void | null () |
|  | Handle the null literal value. More... |
|  | |
| virtual void | boolean (bool p) |
|  | Handle the boolean literal value of the specified polarity. More... |
|  | |
| virtual void | number\_double (double x) |
|  | Handle a the specified double-precision floating point value. More... |
|  | |
| virtual void | number\_long (long n) |
|  | Handle a the specified long integer value. More... |
|  | |
| virtual void | number\_unsigned\_long (unsigned long n) |
|  | Handle a the specified unsigned long integer value. More... |
|  | |
| virtual void | string (const std::string &s) |
|  | Handle the specified string value. More... |
|  | |
| virtual void | key (const std::string &s) |
|  | Handle the specified object key. More... |
|  | |

## Detailed Description

Abstract base class for JSON handlers.

More efficient to just implement directly and pass in as a template, but this version is available for convenience.

Definition at line 15 of file json\_handler.hpp.

## Constructor & Destructor Documentation

|  |  |  |  |  |
| --- | --- | --- | --- | --- |
| stan::json::json\_handler::json\_handler | ( |  | ) |  |

inline

Definition at line 18 of file json\_handler.hpp.

|  |  |  |  |  |
| --- | --- | --- | --- | --- |
| stan::json::json\_handler::~json\_handler | ( |  | ) |  |

inline

Definition at line 20 of file json\_handler.hpp.

## Member Function Documentation

|  |  |  |  |  |  |
| --- | --- | --- | --- | --- | --- |
| virtual void stan::json::json\_handler::boolean | ( | bool | *p* | ) |  |

inlinevirtual

Handle the boolean literal value of the specified polarity.

Parameters
:   |  |  |
    | --- | --- |
    | p | polarity of boolean |

Reimplemented in stan::json::json\_data\_handler.

Definition at line 62 of file json\_handler.hpp.

|  |  |  |  |  |
| --- | --- | --- | --- | --- |
| virtual void stan::json::json\_handler::end\_array | ( |  | ) |  |

inlinevirtual

Handle the end of an array.

Reimplemented in stan::json::json\_data\_handler.

Definition at line 40 of file json\_handler.hpp.

|  |  |  |  |  |
| --- | --- | --- | --- | --- |
| virtual void stan::json::json\_handler::end\_object | ( |  | ) |  |

inlinevirtual

Handle the end of an object.

Reimplemented in stan::json::json\_data\_handler.

Definition at line 50 of file json\_handler.hpp.

|  |  |  |  |  |
| --- | --- | --- | --- | --- |
| virtual void stan::json::json\_handler::end\_text | ( |  | ) |  |

inlinevirtual

Handle the the end of the text.

Reimplemented in stan::json::json\_data\_handler.

Definition at line 30 of file json\_handler.hpp.

|  |  |  |  |  |  |
| --- | --- | --- | --- | --- | --- |
| virtual void stan::json::json\_handler::key | ( | const std::string & | *s* | ) |  |

inlinevirtual

Handle the specified object key.

Parameters
:   |  |  |
    | --- | --- |
    | s | String object key to handle. |

Reimplemented in stan::json::json\_data\_handler.

Definition at line 98 of file json\_handler.hpp.

|  |  |  |  |  |
| --- | --- | --- | --- | --- |
| virtual void stan::json::json\_handler::null | ( |  | ) |  |

inlinevirtual

Handle the null literal value.

Reimplemented in stan::json::json\_data\_handler.

Definition at line 55 of file json\_handler.hpp.

|  |  |  |  |  |  |
| --- | --- | --- | --- | --- | --- |
| virtual void stan::json::json\_handler::number\_double | ( | double | *x* | ) |  |

inlinevirtual

Handle a the specified double-precision floating point value.

Parameters
:   |  |  |
    | --- | --- |
    | x | Value to handle. |

Reimplemented in stan::json::json\_data\_handler.

Definition at line 70 of file json\_handler.hpp.

|  |  |  |  |  |  |
| --- | --- | --- | --- | --- | --- |
| virtual void stan::json::json\_handler::number\_long | ( | long | *n* | ) |  |

inlinevirtual

Handle a the specified long integer value.

Parameters
:   |  |  |
    | --- | --- |
    | n | Value to handle. |

Reimplemented in stan::json::json\_data\_handler.

Definition at line 77 of file json\_handler.hpp.

|  |  |  |  |  |  |
| --- | --- | --- | --- | --- | --- |
| virtual void stan::json::json\_handler::number\_unsigned\_long | ( | unsigned long | *n* | ) |  |

inlinevirtual

Handle a the specified unsigned long integer value.

Parameters
:   |  |  |
    | --- | --- |
    | n | Value to handle. |

Reimplemented in stan::json::json\_data\_handler.

Definition at line 84 of file json\_handler.hpp.

|  |  |  |  |  |
| --- | --- | --- | --- | --- |
| virtual void stan::json::json\_handler::start\_array | ( |  | ) |  |

inlinevirtual

Handle the start of an array.

Reimplemented in stan::json::json\_data\_handler.

Definition at line 35 of file json\_handler.hpp.

|  |  |  |  |  |
| --- | --- | --- | --- | --- |
| virtual void stan::json::json\_handler::start\_object | ( |  | ) |  |

inlinevirtual

Handle the start of an object.

Reimplemented in stan::json::json\_data\_handler.

Definition at line 45 of file json\_handler.hpp.

|  |  |  |  |  |
| --- | --- | --- | --- | --- |
| virtual void stan::json::json\_handler::start\_text | ( |  | ) |  |

inlinevirtual

Handle the the start of the text.

Reimplemented in stan::json::json\_data\_handler.

Definition at line 25 of file json\_handler.hpp.

|  |  |  |  |  |  |
| --- | --- | --- | --- | --- | --- |
| virtual void stan::json::json\_handler::string | ( | const std::string & | *s* | ) |  |

inlinevirtual

Handle the specified string value.

Parameters
:   |  |  |
    | --- | --- |
    | s | String value to handle. |

Reimplemented in stan::json::json\_data\_handler.

Definition at line 91 of file json\_handler.hpp.

---

The documentation for this class was generated from the following file:

- src/stan/io/json/json\_handler.hpp


---

[ Stan Home Page ]

*© 2011–2014,
Stan Development Team.*
